# Preacutionary labelling of cross-reactive foods: The case of rapeseed

**DOI:** 10.1186/s40733-016-0028-4

**Published:** 2016-11-01

**Authors:** Alessandro Fiocchi, Lamia Dahdah, Carla Riccardi, Oscar Mazzina, Vincenzo Fierro

**Affiliations:** Ospedale Pediatrico Bambino Gesù, Dipartimento Pediatrico Universitario Ospedaliero (DPUO), UOC Allergologia, Rome, Vatican City Italy

## Abstract

Food allergic individuals are exposed to unnecessary dietary restrictions due to precautionary food allergy labelling (PFAL). Two forms of PFAL exist: type I identifies the possible presence of allergenic contaminaion in foods (‘may content…’), type II indicates as potentially dangerous ingredients or contaminants that do no belong to official list of food allergens. PFAL type II is based on the fear of cross-reactivity with foods belonging to that list. PFAL type II is less known, but may be tempting for the legal offices of food companies, for clinicians in a ‘defensive medicine’ key, and even for legislators. We identify here a case of PFAL type II, allergy to rapeseed (belonging to the family of *Brassicaceae*). Increasingly used for their nutritional and nutraceutic value in asthma prevention, rapeseed has been indicated by regulatory authorities in Canada and Europe as potential cross-reactor with mustard. In this review, we provide the elements for a risk assessment of cross-reactivity of rapeseed/mustard allergy in the general population both clinically and from the point of view of the molecular allergy. Three findings emerge:

1. Allergic reactions to rapeseed are exceptional

2. The allergens identified in rapeseed and mustard are similar, but not identical

3. Reactions to rapeseed have never been described in mustard-allergic patients.

On the ground of existing evidence, a precautionary labeling for rapeseed as potentially dangerous for patients allergic to mustard is not justified. In the interest of patients with multiple food allergy, PFAL type II must be avoided.

## Background

Labelling is an issue of relevance to food allergic consumers of fresh, processed or pre-packaged foods, as accidental ingestion of food allergens due to labelling ambiguities is a modifiable risk factor. In the European Union, twelve food items are required by law to appear on the label of pre-packaged foods: cereals containing gluten, crustaceans, egg, fish, peanut, soy, milk (including lactose), nuts, mustard, sesame seeds, celery, and sulphites >10 mg/kg [1]. Similar legislation is in effect in the US for only eight foods: milk, egg, peanut, tree nuts, shellfish, fish, soy and wheat [2]. This list do not include mustard, present in the Canada list [3]. The legislation minimizes the risk that unfamiliar names can hide allergenic foods, as for instance “starch” is replaced by “corn starch” or “wheat starch”; “Lysozyme” by “lysozyme, containing egg”, and so on.

On both the sides of Atlantic, the regulatory problem is now the opposite: whether too many foods containing allergenic foods are being labeled as allergenic, and whether this would potentially restrict potentially safe food choices for allergic consumers [4].

Precautionary Food Allergy Labelling (PFAL) has two faces.

The first is the labelling of existent, or not completely excludible, trace amounts of the allergenic foods: no legislation prescribes the indication of potential contaminants, but many manufacturers are now warning of potential contaminations during food preparation. The ways to express such kind of PFAL are so many, that the regulatory authorities in some countries felt the need to regulate this aspect also [5]. This is the case of Canada, where from 2012 the only permitted expression is “may contain [X]” [6]. We will call this PFAL type I.

The labelling of potentially cross-reacting foods (PFAL type II) is another example of potential overlabelling. *Leguminosae* are exempt from this risk in Europe and the Sates, as the indication is very specific for peanut and for soy, so that lentil, pea or bean will not be labeled as ‘potentially cross-reactive’. For milk, the European legislation is clear in indicating that it should be labelled irrespective of its animal origin, reflecting the likely cross-reactivity between cow, sheep, goat, buffalo etc. However, the requirement of labelling lactose as allergenic contradicts the fact that this sugar has never been reported to determine allergic reactions after ingestion among children with CMA [7].

For mustard, it seems clear both in EU and in Canada that this should be the only member of the *Brassicaceae* family to be labeled. Yet, in the explanatory documents, Health Canada warns consumers with mustard allergy to avoid consuming other members of the Brassicaeae family such as broccoli, cauliflower, cabbage, Brussel sprouts, turnip and rapeseed “as these have the potential to trigger an adverse reaction” [8]. In addition, in its Scientific Opinion on the safety of “rapeseed protein isolate” as a novel food ingredient, the European Food Safety Authority reassured on the toxicological aspects, but stated, “…the risk of sensitization to rapeseed cannot be excluded and it is likely that rapeseed can trigger allergic reactions in mustard allergic subjects” [9].

If it comes true, the doctors should be aware of this risk, and the population protected from it. Following the logic of this advice, when an industry is commercializing prepackaged foods containing rapeseed, they must also label them as potentially cross-reactive with mustard. Is this sound, or would it expose allergic individuals to unnecessary restrictions due to PFAL type II [10]?

Thus, the problem is of interest not only of doctors and patients, but also of food industry, drug manufacturers and the regulatory authorities. In this review, we aim to provide the elements for a risk assessment of rapeseed allergy in the general population. We will review the literature to assess, for each molecular allergen, the risk of cross-reactions among rapeseed and mustard.

## Rapeseed

Rapeseed (Brassica napus), also known as rape, oilseed rape, rapa, rappi, and canola [11], is a bright-yellow flowering. The plant is cultivated mainly for its oil-rich seed, the third-largest source of vegetable oil in the world [12]. Rapeseed oil is one of the oldest vegetable oils, but has been used in limited quantities due to high levels of erucic acid, which is damaging to cardiac muscle of animals, and glucosinolates, which made it less nutritious in animal feed. In 1981, a deadly outbreak of disease in Spain, known as toxic oil syndrome, was caused by the consumption of rape oil for industrial use that was fraudulently sold as olive oil to be consumed in cooking, salads, and other foods. Symptoms appeared as a typical pneumonia with interstitial infiltrates on chest X-ray, complicated by pulmonary hypertension in a significant number of cases [13]. Thus, rapeseed oil is subjected to regulatory limitations: the United States Food and Drug Administration recognized rapeseed canola-equivalent oil, also known as ‘low erucic acid rapeseed (LEAR) oil’, as safe. Canola oil is limited to a maximum of 2 % erucic acid by weight in the USA [14] and 5 % in the EU [15]. In 2015–6, the market for rapeseed has gone up considerably and cash market prices exceeded the previous year’s level for several weeks. The upward trend continues since some years [16], for at least two reasons. First, the Food and Agriculture Organization of the United Nations (FAO) encourages the use of novel protein sources (including specifically rapeseed) to reduce the energetic costs of the food production [17]. Second, evidence is emerging that *Brassicaceae* may exert an asthma-preventing action. For instance, the consumption of kimchi – a traditional fermented Korean side dish made from napa cabbage and radish – is inversely associated with the incidence of asthma [18]. Broccoli have been shown to activate NK cells during asthma-inducing respiratory infections [19]. Their consumption is being proposed as a nutritional antioxidant intervention for reducing influenza risk among populations at risk of asthma [20]. Cataplasms of white mustard seed, traditionally used in China to prevent asthma, are currently studied for their mechanisms of action [21]. Thus, we may expect an increase of the use of *Brassicaceae* as foods or dietary supplements. This could increase the odds of allergic reactions [22].

## Rapeseed allergens

Rapeseed pollen contains known allergens (Table 1), but rape pollen do not causes hay fever, as rape is an insect-pollinated (entomophilous) crop, whereas hay fever is usually caused by wind-pollinated plants. The inhalation of rape dust may cause asthma in agricultural workers. As rapeseed is consumed as an oil, containing only little amounts of protein allergens, rapeseed food allergy is practically an unknown [23]. Hereafter, we will indicate the single rapeseed potential food allergens.

**Table 1:** rapeseed allergens and their IUS code

Allergen	Name	Code
Bra n	Napin	1750
Bra n 1	Napin	169
Bra n 1.0101	Napin	3162
Bra n 4	Polcalcin	710
Bra n 7	Polcalcin	170
Bra n 8	Profilin	1073
Bra n PG	Polygalactonurase	1072
	Cruciferin	nf

### Bra n 1 (Napin n III)

Napins have been identified as rapeseed storage proteins in the ‘90s, but have been characterized as allergens only recently. Proteomic methods indicate that these protein allergens accumulate during seed development [24]. Napins represent approximately 20 % of the total protein in rapeseed flour. They consist of a small and large chain linked by disulphide bonds [25], resistant to pepsin digestion and denaturation caused by heat and low pH [26]. Thus, it is possible that napin is not destroyed during conventional food processing.

Napins are ligands of sIgE, as shown by IgE immunoblotting and IgE ELISA in the sera from 72 atopic children with positive SPT responses to oilseed and turnip rape. They are a group of homologous proteins belonging to the family of 2S albumins, MW 9.5- to 14.5-kd. About 80 % of the patients had IgE to purified napins from both plants at ELISA. In SPTs purified napins caused positive reactions in all children tested [27]. These allergens seem more frequently positive in Finnish children with atopic dermatitis (AD) than in French AD children. IgE antibodies to purified 2S albumin allergens showed cross-wise IgE inhibition patterns among turnip rape, oilseed rape and mustard seeds. Thus, the 2S albumin allergens in the seeds of these plants are highly cross-reactive and could be important sensitizers in children with atopic dermatitis [28].

### Bra n 4 – calcium – binding protein, polcalcin

Polcalcins are a family of pollen allergens identified in several evolutionarily distant dicotyledon and monocotyledon plants. They belong to the family of calcium-binding proteins, characterized by a variable number of motifs, termed EF-hands, which consist of two perpendicularly placed alpha-helics and an inter-helical loop forming a single calcium-binding site. Due to their ability to bind and transport calcium as well as to interact with a variety of ligands in a calcium-dependent manner, they fulfill important biological functions in eukaryotic cells. Polcalcinis specifically are EF-hand proteins believed to assist in regulating pollen-tube growth (*see infra*). After parvalbumin, a three EF-hand fish allergen, calcium-binding allergens were discovered in pollens of trees, grasses and weeds and, recently, also as autoallergens in man. Although only a small percentage of atopic individuals displays IgE reactivity to calcium-binding allergens, these allergens may be important because of their ability to cross-sensitize allergic individuals. Confrontation and stability, as well as IgE recognition of calcium-binding allergens, greatly depend on the presence of protein-bound calcium ions. It is thus likely that hypoallergenic derivatives of calcium-binding allergens can be engineered by recombinant DNA technology for immunotherapy of sensitized patients [29].

Polcalcins were identified in 1995 as allergens in the anthers of Brassica rapa L. and B. napus L. using the serum IgE from a patient specifically allergic to Brassica pollen. At that time, botanicals were able to identify genotypic and phenotypic sequences of a group I polcalcin of 79 amino acids, and a group II polcalcin of 83 amino acids, with microheterogeneity [30]. Later on, Bra – 4, a polcalcin, has been identified as an inhalant allergen from the pollen of Brassica. This is a 8.6-kD protein with two EF-hand calcium-binding motifs, member of a small gene family in Brassica napus. Homologs have been detected in Arabidopsis, from which one gene has been cloned, and in snapdragon (Antirrhinum majus). Expression of the gene in B. napus was limited to male tissues and occurred during the pollen-maturation phase of anther development. Both the B. napus and Arabidopsis proteins interact with calcium, and the potential for a calcium-dependent conformational change was demonstrated. Given this affinity for calcium, the cloned genes were termed BPC1 and APC1 (B. napus and Arabidopsis pollen calcium-binding protein 1, respectively). Immunolocalization studies demonstrated that BPC1 is found in the cytosol of mature pollen. However, upon pollen hydration and germination, there is some apparent leakage of the protein to the pollen wall. BPC1 is also concentrated on or near the surface of the elongating pollen tube. The essential nature of calcium in pollen physiology, combined with the properties of BPC1 and its high evolutionary conservation, suggests that this protein plays an important role in pollination by functioning as a calcium-sensitive signal molecule [31].

### Bra n 7 – calcium – binding protein, polcalcin

Another polcalcin has been identified as allergen in rapeseed by immunoblot, immunoblot inhibition and specific monoclonal antibodies using sera from 89 patients sensitized to rapeseed. Two low-molecular-weight allergens of 6/8 kD were identified in 50 % of patients, totally cross-reactive with rye pollen and moderately with birch pollen. Binding to the 6/8-kD rape allergen could be effectively inhibited by rAln g 2, a calcium-binding protein from alnus. These data demonstrated that this little calcium-binding protein, thinner than Bran 4, is an important respiratory allergen of rapeseed [32]. It exhibits wide cross-reactivity with tree pollens, thimothy grass, and herbs [33].

### Bra n 8 – actin–binding protein, profilin

Allergenic plant profilins constitute a highly conserved family with sequence identities of 70 to 85 % among each other but low identities of 30 to 40 % with nonallergenic profilins from other eukaryotes, including human beings. Profilins are present as food allergens in a variety of fruits and vegetables, and have been associated with non-anaphylactic reactions. In rapeseed, they were found at immunoblot, immunoblot inhibition and/or specific monoclonal antibodies in 34 % of sensitized patients [12]. An anti-profilin-specific monoclonal antibody bound specifically to this 14-kD protein. Later on, the 14-kD protein, purified from the other allergens, was precisely identified by PLP affinity chromatography as a single protein of 14.5 kD and further identified as a profilin by three polyclonal rabbit antisera. It is very similar to ragweed and tobacco pollen profilin and the C-terminus of birch profilin. Skin prick tests were positive with Bra n 8 in two thirds of patients [34].

### Bra n PG – polygalactonurase

The third group of proteins identified as allergenic in oilseed rape is a large group of 27 to 69-kD allergens, rich in carbohydrate determinants [12]. This cluster is cross-reactive with grass pollen group 4 allergens. They peak at a molecular mass around 43 kD. From this zone, two isoforms of the polygalacturonase enzyme were identified by microsequencing, confirmed by MALDI-TOF mass spectrometry analysis. As 80 % of rapeseed sensitive patients show reactivity to this cluster, Bra n PG seems to be the main allergen for some patients [35].

### Cruciferin

The main seed storage protein in these plants is an 11S globulin, cruciferin, a large, neutral, oligomeric protein synthesized in rapeseed during the seed development. It is composed of six subunit pairs. Each pair consists of one heavy α chain (30 kDa) and one light β chain (20 kDa). Four different subunit pairs exist. Investigations using two-dimensional electrophoresis showed that the majority of α and β chains of each subunit are disulfide-linked [36]. Cruciferin is one of the major allergens also in white mustard [37] and hazelnut [38].

## Prevalence of allergy to rapeseed

IgE-mediated food allergy to members of the *Brassicaceae* family is uncommon [39–41], except for mustard [42–44]. Rapeseed allergy been described among rapeseed workers by contact and/or inhalation of rapeseed proteins [45], but rapeseed food allergy is very rare, so that no specific epidemiologic study has been dedicated to it. Despite its wide consumption in China and Africa, no single case of allergic reaction to rapeseed flour or oil can be found in the literature in that countries. However, its ability to determine clinical reactions has been demonstrated in experimental models (*see infra).*

Regarding rapeseed sensitization, children with IgE-mediated allergy to vegetable foods may react to seeds of oilseed rape and turnip rape in skin prick tests (SPTs), but no studies have formally addressed the specific question in open populations. A Finnish study among children with atopic dermatitis suspected for food allergy showed that 11 % (206/1887) of such children were SPT-positive to oilseed (Brassica napus) and/or turnip (Brassica rapa) rape [46]. None of them had immediate or delayed symptoms from this sensitization. A further study examined 64 children with atopic dermatitis and a positive skin prick test to turnip rape and/or oilseed rape. These were found polysensitized, with positive skin prick tests reactions and IgE antibodies to various foods (cow’s milk, egg, wheat, mustard) and pollens (birch, timothy, mugwort). They had often associated asthma (36 %) and allergic rhinitis (44 %). Children with atopic dermatitis sensitized to oilseed rape and turnip rape had high frequency of associated sensitizations to all foods and pollens tested, showing that oilseed plant sensitization affects especially atopic children who have been sensitized to multiple allergens. In other words, this seems a sensitization without clinical correlates.

To characterize the clinical significance of these sensitizations, *i.e.* the ability of rapeseed flour to determine allergic reactions, 28 children with clearly positive SPT (> or =5 mm) were subjected to labial challenge with turnip rape seeds followed, if negative, by open oral challenge for up to 7 days. Twenty-five (89 %) of the 28 showed a positive challenge reaction to turnip rape. Seventeen reacted with labial whealing, and eight in oral challenge with facial urticaria, flare-up of AD or abdominal symptoms. This indicates that turnip rape and oilseed rape may act as allergy triggers in young children with AD [47]. Later on, food challenges with turnip rape and mustard were performed to 14 Finnish and 14 French children with atopic dermatitis and positive skin prick test to turnip rape. Open labial or oral challenge to turnip rape was positive in 14 (100 %) Finnish and five (36 %) French children and mustard challenge in five Finnish and five French children [8]. These findings indicate that turnip rape flour may act as an allergen and may cross-react with mustard, but the scarcity of the exposed data makes impossible to estimate the frequency of sensitization in different geographical areas. This is even truer outside Europe, where epidemiological studies on food allergy are practically absent. That said, the majority of rapeseed is consumed as oil, available as cold-pressed or refined oil. Refined oils do not cause allergic reactions, as they do not contain proteins. During the manufacture of cold-pressed oils, however, oil is pressed from the oilseeds without heating and the sediment from the seeds is settled down. Thus these kinds of oils may contain traces of seed proteins. Allergenic napins (2S albumin) and allergenic cruciferins (11S globulin) can be detected in cold-pressed rapeseed oils with sensitive proteomic methods [48]. However, the clinical importance of the exposure to low concentration such allergens in rapeseed oil is not known.

## Potentially dangerous thresholds of contamination

As probably no clinical reaction to rapeseed has even been described, it is impossible to express thresholds for a risk of allergic reactions. Reviewing the data on threshold levels for food allergic reactions, a strong interindividual variability appears immediately. The calculated dose of fatal anaphylaxis to peanut (one of the most frequent event) varies from few milligrams to several tens of grams [49]. The interindividual threshold levels differ substantially and it is difficult to reach general recommendations for allergic subjects [50]. To overcome these limits, recent studies afforded the topic at a European-wide level, with the aim to describe the spectrum of provoking doses for food allergy and ultimately to assist the European Community in their legislation [51]. No specific evaluation of rapeseed allergy has been included.

## Cross-reactivity among rapeseed and mustard seed

Anaphylactic reactions set off by the ingestion of a small amount of mustard sauce are described since longtime [52]. Several different mustard species are commonly used in food: white mustard (Sinapis alba), black mustard (Brassica nigra) and brown mustard (Brassica juncea) are the most common [53]. White mustard is detected in the “green” and black/brown mustard in the “yellow” channel. In a prospective study on mustard allergic patients, 38 patients (10.5 % reporting systemic anaphylaxis) showed association with mugwort pollen sensitization (97.4 %) and other members of *Brassicaceae* family. Associations with nut (97.4 %), leguminosae (94.7 %), corn (78.9 %), and Rosaceae (89.5 %) sensitizations were also reported. Around 40 % of these food sensitizations were symptomatic, including food-dependent exercise-induced anaphylaxis in six patients. These data prompted the definition of ‘mustard-mugwort allergy syndrome’ [54], and allergens in mustard seed have been extensively studied (Table 2 and Table 3).

**Table 2:** Mustard seed allergens, their origin and their IUS code

Allergen	Source	Code
Bra ni	Black Mustard, Brassica nigra	1682
Bra j	Brassica juncea, Oriental Mustard	1749
Bra r	Bird Rape, Brassica rapa, Field Mustard, Turnip, Turnip Mustard	2022
Sin a	Bird Rape, Brassica alba, Sinapis alba, Turnip, White Mustard	1972
Bra j 1	Brassica juncea, Oriental Mustard	168
Bra r 1	Bird Rape, Brassica rapa, Field Mustard, Turnip, Turnip Mustard	2682
Bra r 3	Bird Rape, Brassica rapa, Field Mustard, Turnip, Turnip Mustard	1060
Sin a 1	Bird Rape, Brassica alba, Sinapis alba, Turnip, White Mustard	627
Sin a 1.0101	Bird Rape, Brassica alba, Sinapis alba, Turnip, White Mustard	3477
Sin a 1.0104	Bird Rape, Brassica alba, Sinapis alba, Turnip, White Mustard	1662
Sin a 1.0105	Bird Rape, Brassica alba, Sinapis alba, Turnip, White Mustard	1663
Sin a 1.0106	Bird Rape, Brassica alba, Sinapis alba, Turnip, White Mustard	1664
Sin a 1.0107	Bird Rape, Brassica alba, Sinapis alba, Turnip, White Mustard	1665
Sin a 1.0108	Bird Rape, Brassica alba, Sinapis alba, Turnip, White Mustard	1666
Sin a 2	Bird Rape, Brassica alba, Sinapis alba, Turnip, White Mustard	2693
Sin a 2.0101	Bird Rape, Brassica alba, Sinapis alba, Turnip, White Mustard	4034
Sin a 3	Bird Rape, Brassica alba, Sinapis alba, Turnip, White Mustard	7634
Sin a 3.0101	Bird Rape, Brassica alba, Sinapis alba, Turnip, White Mustard	7635
Sin a 4	Bird Rape, Brassica alba, Sinapis alba, Turnip, White Mustard	7636
Sin a 4.0101	Bird Rape, Brassica alba, Sinapis alba, Turnip, White Mustard	7637

**Table 3:** If a company will label rapeseed as possible cross-reactor with mustard, they should also label [63–70]

…Celery	as possibly cross-reactive with….	… anise, fennel, coriander, cumin, caraway, carrot, dill, lovage, parsley…
…Cereals containing gluten		… triticale, rye, barley, oat, rice, maize
…Egg		… poultry
…Peanut		… beans, lentils, lupin, chick pea….
…Nuts		… peach, apricot, kiwifruit…
…Soy		… peas, lentils, kidney, lima and navy beans…
…Milk		… beef
…Mustard		… rapeseed, sunflower seed, ricinus ….

**Bra ni** and **Bra r** do not have a specific literature. **Bra j** is a specific allergen of the Oriental mustard, a food available also in transgenic form (Brassica juncea expressing choline oxidase gene from Arthrobacter globiformis that provides resistance against abiotic stresses) [55]. The major allergenic protein in yellow mustard (Sinapis alba) is the 2S napin **Sin a 1**. It shows no cross reactivity with other proteins of mustard or other Brassicaceae proteins of similar genetic homology [56]. **Sin a 2**, a 11S globulin, has been characterized as a major 51 kDa allergen. The allergen was dissociated in 2 chains of 36 and 23 kDa, which also bound IgE. The common basis is a seed storage 11S globulin belonging to the Cupin super family. It has been associated with severe allergic reactions and is involved in cross-reactivity at the IgE level with tree nuts and peanut. *Brassicaceae* do have cruciferin and Lipid-Transfer Protein (LTP), able to act as allergens and even to determine severe reactions (as Bra o 3 in patients allergic to Cabbage [57]).

The mustard LTP is **Sin a 3**, whose specific cDNAs has been identified and encodes for a mature proteins of 92 amino acids. Sin a 3 shows 54 % identity with allergenic LTP from peach. **Sin a 4** specific DNA has been also sequenced, encoding for a protein of 131 amino acids that belong to profilin family. Sin a 4 showed 80 % in - vitro identity with allergenic profilin from melon [58].

In vitro studies showed cross-reactivities among mustard and other vegetables, such as nuts, peach, and melon, and other *Brassicaceae*. From the analysis of the examined studies, one can conclude than – although cross-sensitization cannot be excluded – no cross-reactivity has been demonstrated among mustard and rapeseed in vivo at inhalant and food allergy level.

## Conclusions

In a time when new allergenic foods are described every year, there is concern in the public opinion about the possibility of hidden allergens in processed foods [59]. The consumers are also exposed to uncontrolled lay information on the possible risks of ‘new’ food allergies. Recent examples show how this can translate in the creation of social alarm, for disparate reasons [60]. The implementation of PFAL type II could add to their panic, but would be necessary if cross-reactivity are frequent as in the case of milk.

In our case, a cross – reaction among rapeseed and mustard is theoretically possible but it has never been documented. In this situation, is it opportune to label a food as potentially cross-reactive on the ground of a theoretical possibility only? We fear that this would add confusion among the consumers and health professionals who provide them with advice on how to manage their condition [61, 62]. Allergists spend the majority of their clinical work to prevent unnecessary elimination of foods, and PFAL type II would contradict their efforts.

If the contrary would apply, in the context of the pre-packed snacks production at least the alerts indicated in Table 2 should be activated [63–70]: we have no doubt that this would worsen the quality of life of allergy sufferers.
